# Characterization of Ferroelectric *Al*_0.7_*Sc*_0.3_*N* Thin Film on Pt and Mo Electrodes

**DOI:** 10.3390/mi13101629

**Published:** 2022-09-28

**Authors:** Ran Nie, Shuai Shao, Zhifang Luo, Xiaoxu Kang, Tao Wu

**Affiliations:** 1 Shanghai Engineering Research Center of Energy Efficient and Custom AI IC, School of Information Science and Technology, ShanghaiTech University, Shanghai 201210, China; 2 Shanghai Institute of Microsystem and Information Technology, Chinese Academy of Sciences, Shanghai 200050, China; 3 University of Chinese Academy of Sciences, Beijing 100049, China; 4 Process Technologies Department, Shanghai IC R&D Center, Shanghai 201203, China

**Keywords:** AlScN, ferroelectric, thin film, leakage current, PUND test

## Abstract

In the past decade, aluminum scandium nitride (AlScN) with a high Sc content has shown ferroelectric properties, which provides a new option for CMOS-process-compatible ferroelectric memory, sensors and actuators, as well as tunable devices. In this paper, the ferroelectric properties of $Al_{0.7}Sc_{0.3}N$ grown on different metals were studied. The effect of metal and abnormal orientation grains (AOGs) on ferroelectric properties was observed. A coercive field of approximately 3 MV/cm and a large remanent polarization of more than 100 μC/cm^2^ were exhibited on the Pt surface. The $Al_{0.7}Sc_{0.3}N$ thin film grown on the Mo metal surface exhibited a large leakage current. We analyzed the leakage current of $Al_{0.7}Sc_{0.3}N$ during polarization with the polarization frequency, and found that the $Al_{0.7}Sc_{0.3}N$ films grown on either Pt or Mo surfaces have large leakage currents at frequencies below 5 kHz. The leakage current decreases significantly as the frequency approaches 10 kHz. The positive up negative down (PUND) measurement was used to obtain the remanent polarization of the films, and it was found that the remanent polarization values were not the same in the positive and negative directions, indicating that the electrode material has an effect on the ferroelectric properties.

## 1. Introduction

Ferroelectrics are materials that possess spontaneous polarization in the absence of an applied electric field, and the direction of its polarization vector can be flipped by the applied electric field [1,2]. It is an essential component in a wide range of applications, such as non-volatile memories and radio frequency (RF) devices [3,4,5]. Components based on ferroelectric thin films are also being developed for a variety of sensor and actuator applications, as well as tunable microwave circuits [6,7]. Many ferroelectric materials are perovskites with drawbacks, such as low quasi-electric transition temperatures, nonlinear shifts or limited compatibility with complementary metal oxide semiconductors (CMOSs) or III-nitride technologies. These issues have so far prevented the popularization of ferroelectric functionality in microelectronics [8].

**Figure 1:**
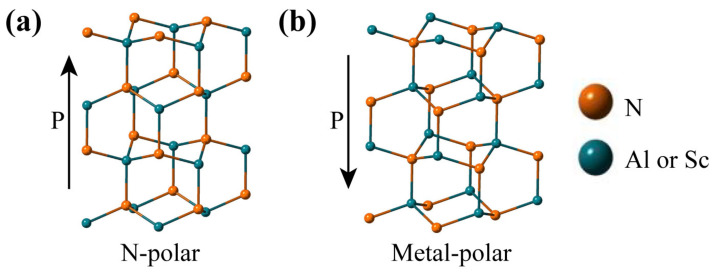
The two polarization states formed when the scandium atom occupies the position of the aluminum atom: (**a**) N-polar and (**b**) metal-polar.

Aluminum nitride (AlN) thin films have a relatively high acoustic phase velocity, low acoustic wave loss, considerable piezoelectric coupling constant, and a coefficient of thermal expansion similar to that of Si and GaAs. These unique properties of AlN films make them widely used in mechanical, microelectronic, optical, MEMS transducers, surface wave devices (SAWs) and high-frequency broadband RF filters in the communication front-end [9,10]. AlN thin film is a III-V group semiconductor with a Wurtzite-type structure, and possesses polarization (N-polar and metal-polar) along the c-axis due to the separation of aluminum and nitrogen atoms in each plane under certain stress conditions [8]. However, AlN does not possess ferroelectricity because its polarization direction cannot be switched in an electric field below its own dielectric breakdown limit. In recent years, aluminum scandium nitride (AlScN) has become a hot research topic [11,12]. The significantly higher piezoelectric coefficient of AlScN compared to AlN has led to piezoelectric devices based on AlScN with high electromechanical coupling coefficients [13]. More interestingly, AlScN has been approved to be ferroelectric with a high Sc ratio, and its ferroelectric switching voltage can be flexibly adjusted depending on the Remanent stress and Sc content to meet the needs of ferroelectric thin films in a wide range of application scenarios [14,15]. The two polarization states of AlScN are shown in Figure 1.

In this work, the ferroelectric properties of AlScN with a 30% Sc content on Pt and Mo bottom electrodes were studied. It was found that the ferroelectric properties of the films grown on Mo and Pt are significantly different. The leakage current of the Mo sample is quite high and the polarization value obtained from the hysteresis curve is almost double that of the Pt sample. In the PUND test, this gap was maintained and it was observed that the remanent polarizations in the positive and negative directions were not equal. Given that the top and bottom electrodes of the device are not of the same material, it can be concluded that different metal electrodes play a role in the ferroelectricity of the film [16].

## 2. Fabrication and Experiment Setup

**Figure 2:**
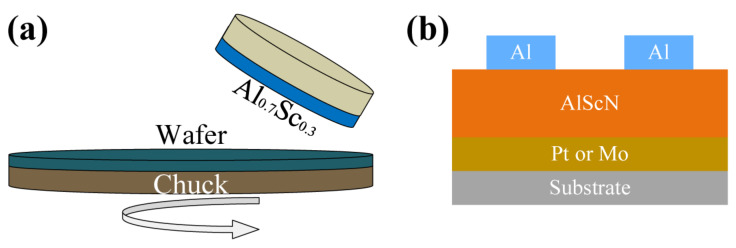
(**a**) Schematic diagram of magnetron sputtering deposition of $Al_{0.7}Sc_{0.3}N$. (**b**) Stacking schematic of Pt/Mo-AlScN-Al structure.

According to previous studies, the AlScN films with Sc contents below 27% are prone to break down near the coercive field, and a distinct ferroelectric polarization occurs with a Sc content of more than 27%. As the Sc contents increase, the coercivity, saturation polarization and remanent polarization all decrease [8,11,14]. Therefore, we prepared AlScN thin films at a specific Sc content of 30% in this study. In the study, 200 nm thick $Al_{0.7}Sc_{0.3}N$ films were deposited using a pulsed DC magnetron reactive sputtering (EVATEC CLUSTERLINE ® 200 MSQ) with a single 4-inch $Al_{0.7}Sc_{0.3}N$ alloy target, as shown in Figure 2a. The films deposited in this way grow along the *c*-axis direction [17,18]. In order to apply the electric field across the film thickness direction, 100 nm Pt and 200 nm Mo were used as the bottom electrodes, respectively. Then, 100 nm Al was used as the top electrodes for a simplified process flow. The final device structure is shown in Figure 2b.

**Figure 3:**
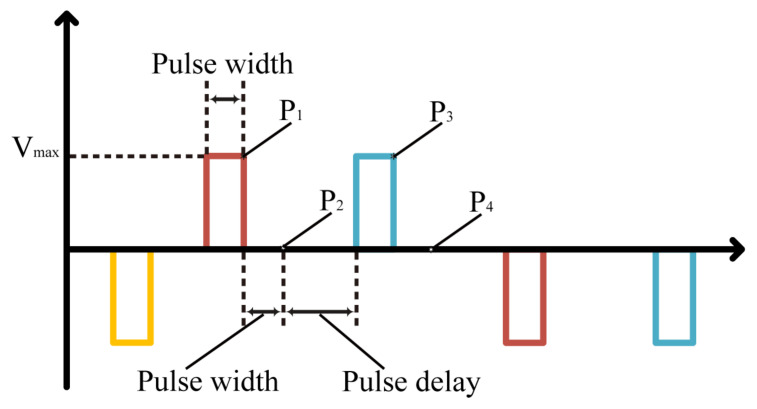
Schematic of the pulse sequence for the PUND test. The pulse intensity is $V_{max}$, and each pulse maintains a pulse width. When a pulse ends, a pulse width plus pulse delay is waited for in order to input the next pulse. The polarization values are recorded twice for each pulse cycle.

X-ray diffraction (XRD) was used to characterize the crystalline quality of $Al_{0.7}Sc_{0.3}N$. A comparison and evaluation of the number of abnormal orientation grains on the film surface was obtained from SEM images. Then, the dielectric properties were measured using Keysight B1500 to test the I−V and C−V curves of the samples. To characterize the ferroelectric polarization of the films in Pt and Mo, hysteresis tests with different polarization voltage and frequencies were performed using a Radiant Multiferroic II II tester. However, other components of the system, including electrodes, leads and interfaces, could dominate the electrical response rather than the intrinsic properties of the material of interest [19]. Therefore, PUND measurements were used to separate the different components of the electrical response of a ferroelectric film. In this measurement, a sequence of five pulses was introduced. The first pulse (pulse 1) flips the polarization of the sample to a defined state. The second pulse is in the opposite direction of the first pulse and $V_{max}$ is maintained for one pulse width to ensure that the sample is saturated with polarization, at which point, the polarization value $P_{1}$ is recorded. After the second pulse, their is a wait of one pulse width and the second polarization value $P_{2}$ is recorded. After certain pulse delay, a third pulse is applied and the third polarization value $P_{3}$ is recorded at its end. Then, there is a wait of one pulse width to record the polarization value $P_{4}$ and subtract $P_{1}$ from $P_{3}$ to obtain dP, since $P_{1}$ contains both switching and non-switching components, whereas $P_{3}$ contains only non-switching components, so dP can represent the correct remanent polarization. Typically, $P_{2}$-$P_{4}$ is written as $dP_{r}$, since $P_{2}$ and $P_{4}$ are the polarization values recorded after waiting for a pulse width and losing a certain polarization, and $dP_{r}$ should be equal to dP. Pulse 4 and pulse 5 are similar to pulse 2 and pulse 3, only in the opposite direction, in order to obtain −dP and $-dP_{r}$. Figure 3 shows the pulse sequence of the PUND test.

## 3. Results and Discussions

### 3.1. Film Quality Characterization

**Figure 4:**
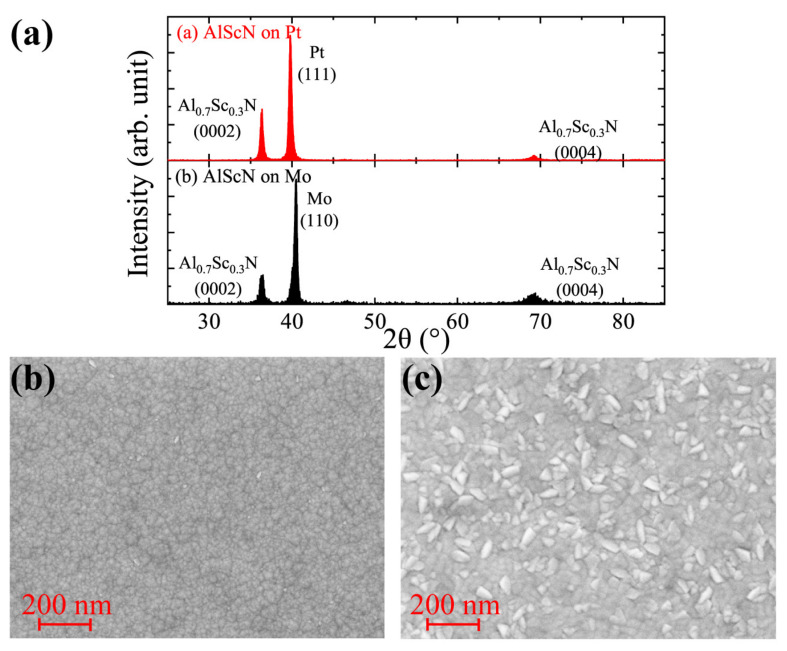
(**a**) X-ray diffraction 2θ/θ scan $Al_{0.7}Sc_{0.3}N$ on (**a**) Pt and (**b**) Mo. The peak of $Al_{0.7}Sc_{0.3}N$ (0002) on the Pt sample is stronger than that of the Mo sample. SEM image of $Al_{0.7}Sc_{0.3}N$ grown on: (**b**) Pt with a good crystal orientation; (**c**) Mo with a large number of abnormal grains.

As shown in Figure 4a, the full width half maximum (FWHM) of the 2θ/θ scans of the 200 nm $Al_{0.7}Sc_{0.3}N$ films grown on Pt and Mo surfaces were 0.36${}^{\circ }$ and 0.50${}^{\circ }$, respectively. The FWHM of the ω-rocking curve of $Al_{0.7}Sc_{0.3}N$ (0002) peak is below 2.5${}^{\circ }$ on Pt samples, and approximately 3${}^{\circ }$ on Mo samples. The absence of other peaks near the $Al_{0.7}Sc_{0.3}N$ (0002) peak indicates that the film has a good c-axis orientation [20]. The diffraction peak of the sample on the Pt surface is stronger and has a better grain orientation [21]. Comparing SEM images Figure 4b,c, there are a large number of abnormal grains of $Al_{0.7}Sc_{0.3}N$ grown on the Mo surface. These AOGs lead to a partial or total loss of the c-axis texture in the surface layer of the films. Since the polarization direction of AlScN is along the c-axis, it can be assumed that the ferroelectric properties of the Mo sample will be worse than those of the Pt sample, which will be further verified in the later measurement.

### 3.2. Dielectric Properties Measurement

**Figure 5:**
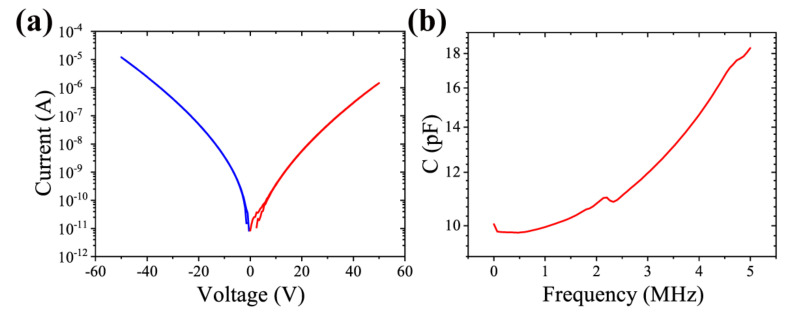
$Al_{0.7}Sc_{0.3}N$ with Pt as the bottom electrode. (**a**) I−V curve with voltage scanning from 0 V to 50 V and then returning to 0 V, similar in the negative direction. (**b**) The C−F scan goes from 1 kHz to 5 MHz and back.

We performed I−V and C−F tests on Pt samples. The scanned voltage from the I-V test does not exceed the coercivity field of the film in order to observe the leakage current of the device. The leakage current of the Pt sample is small as can be seen in Figure 5a. The currents in the positive and negative directions are not symmetrical, which is caused by the Schottky contact between Pt and AlScN and the ohmic contact between Al and AlScN. [22] As shown in Figure 5b, in the C-F test, the frequency was scanned from 1 kHz to 5 MHz, the measured capacitance value increased from 10 pF to 18 pF, and the area of the device under the test was $4\times 10^{-4}$ cm^2^.

### 3.3. Effect of Different Electrodes on P-E Ferroelectric Hysteresis

**Figure 6:**
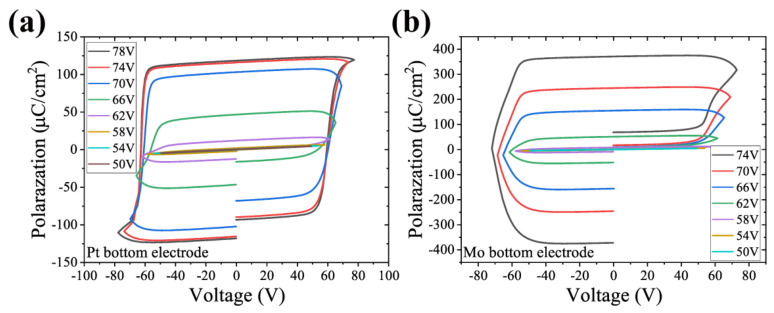
Hysteresis lines of $Al_{0.7}Sc_{0.3}N$ on two electrodes at different voltages. (**a**) Pt samples, with maximum remanent polarization, were approximately 100 μC/cm^2^ and (**b**) Mo samples were approximately 350 μC/cm^2^.

As shown in Figure 6a,b, AlScN has typical ferroelectric properties. The maximum applied drive voltage ranges from 50 V to 82 V in steps of 4 V. The maximum voltage of the Pt sample was only up to 78 V. The sample was broken down after the voltage was increased to 84 V, whereas the Mo sample was broken down at 78 V. As can be seen from the hysteresis loop, the coercive field is approximately 3 MV/cm. However, the samples with two different bottom electrodes show completely different leakage behavior. The $Al_{0.7}Sc_{0.3}N$ films grown on Mo have a large leakage current during the negative polarization. We speculate that this is due to the poor crystal quality and the huge amount of abnormal orientation grains, as the SEM image shows. Comparatively, the Pt sample also exhibits current asymmetry, but very weakly. The remanent polarizations of the two samples obtained from the hysteresis line test were approximately 100 μC/cm^2^ and 350 μC/cm^2^, respectively.

**Figure 7:**
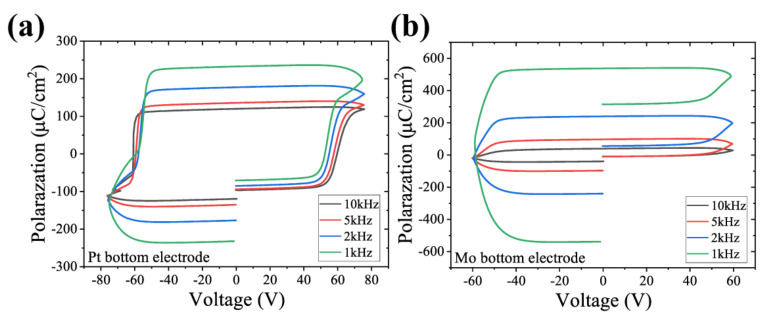
The hysteresis P-E loops with different frequencies of the $Al_{0.7}Sc_{0.3}N$ on (**a**) Pt—as the frequency increases, the polarization flips incompletely and gradually becomes symmetrical—and (**b**) Mo—always asymmetrical.

In order to further analyze this leakage behavior, we measured the hysteresis loops at different frequencies and set the drive voltage just beyond the coercive field. For the Pt sample, ignoring the “gap” caused by electrode asymmetry, the P-E curves in the range of 1 K to 10 K show near standard ferroelectricity, as shown in Figure 7. When the frequency reaches 10 K, there is almost symmetry. However, the P-E curve of the Mo sample is not so good. Although the frequency is increased to a relatively high level so that the polarization does not switch repeatedly, it still exhibits significant asymmetry.

**Figure 8:**
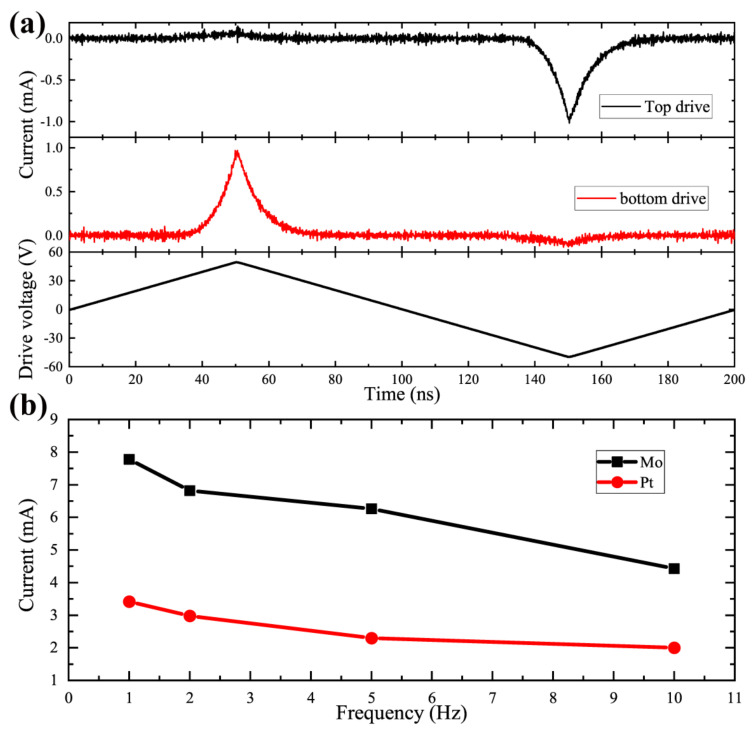
(**a**) Switching current with top and bottom drive under maximum 50 V triangle driving voltage. (**b**) The maximum voltage current as a function of driving frequency with the maximum voltage set to 60 V.

Since the Mo sample exhibited asymmetry, we swapped the drive and sense terminals and obtained the electrical response as shown in Figure 8a. The maximum voltage applied was 50 V, which does not exceed the coercivity field, so the resulting current contains only the leakage current component and not the polarization current. The results show that there is a large leakage current of 1 mA in the negative direction only. Such a phenomenon could be attributed to different electrode materials, as well as asymmetric polarization hysteresis. Once the driving voltage exceeds the coercive field, the large leakage current on the Mo sample causes the hysteresis loop to completely deform, as illustrated in Figure 7b.

In Figure 8b, the peak currents of negative polarization at different frequencies are compared by extracting at 60 V. The measured current of the Mo sample is around 8 mA at a 1 kHz frequency, while showing currents over two times that of the Pt at all frequencies. Such a large leakage current will make the Mo electrode sample easier to break down when its polarization is reversed. On the other hand, the large leakage current makes it possible to output a stronger signal during polarization reversal, which greatly reduces the possibility of a loss of reading.

### 3.4. PUND Test to Obtain the Remanent Polarization

A PUND test was performed to further analyze the ferroelectric properties of $Al_{0.7}Sc_{0.3}N$ on both metals. As mentioned earlier, the main conditions that can be changed in the PUND test are $V_{max}$, pulse width and pulse delay. $V_{max}$ just needs to be large enough to ensure that the polarization can switch. Therefore, we only changed the pulse width and pulse delay to see how the remanent polarization of the device changes.

**Figure 9:**
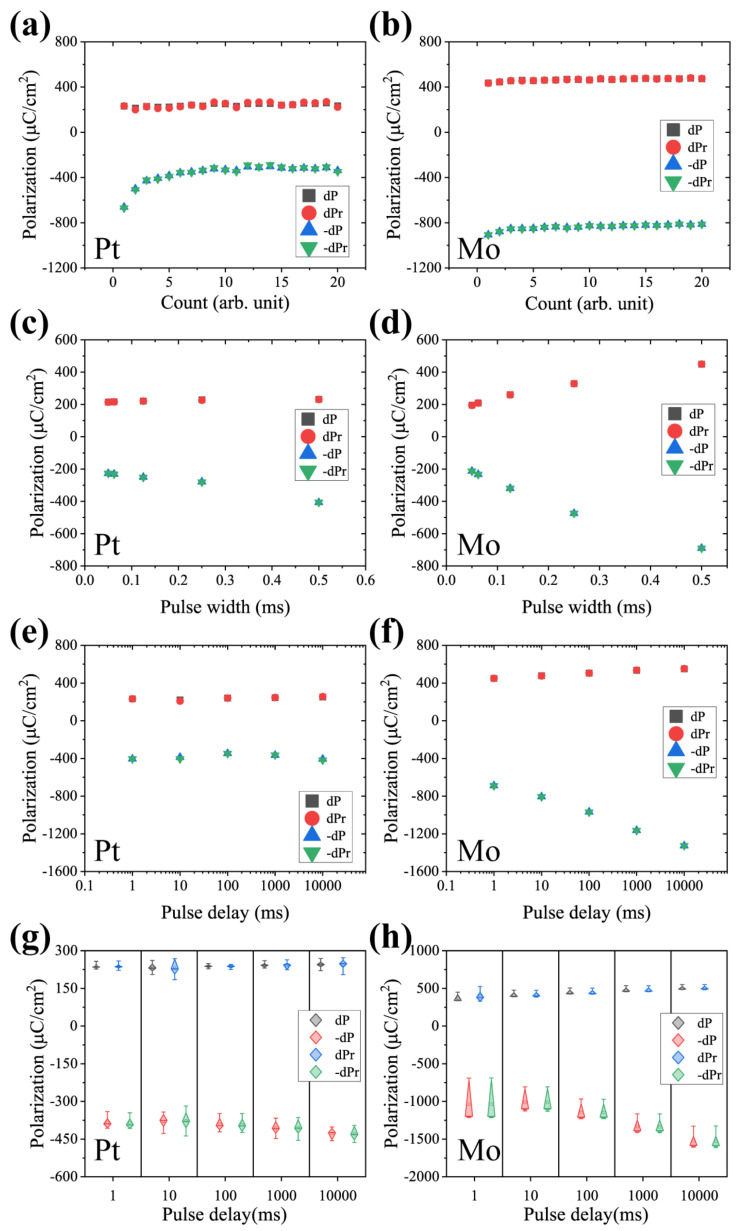
PUND measurements under different conditions. Repeat test at fixed $V_{max}$ = 60, V, pulse width = 0.5 ms and pulse delay = 10 ms: (**a**) Pt and (**b**) Mo. $V_{max}$ and pulse width were kept constant: 60 V and 0.5 ms, pulse delay set to 1, 10, 100, 1000 and 10,000 ms. (**c**) Pt and (**d**) Mo. Pulse width was taken as 0.5, 0.25, 0.125, 0.625 and 0.05 ms. In addition, $V_{max}$ = 60 V, pulse delay = 1 ms. (**e**) Pt and (**f**) Mo. Distribution of PUND results for different samples at pulse wide was 0.5 ms, pulse delay changed from 1 ms to 10,000 ms, (**g**) Pt and (**h**) Mo.

First, $V_{max}$ was set to 60 V, pulse width to 0.5 ms, pulse delay to 10 ms and each test was subjected to 20 repetitions of the experiment, as shown in Figure 9a,b. An interesting phenomenon appears here: the remanent polarization in the negative direction of the Pt sample is larger for the first few times of the power-up test after resting at one end of the time, and then gradually decreases and stabilizes. This may be due to some parasitic parameters, which are subject to further analysis. In addition, the remanent polarization of the Pt sample is around 200 μC/cm^2^ in the positive direction and 260 μC/cm^2^ in the negative direction, a difference brought about by the Schottky contact between Pt and AlScN. The polarization of the Mo sample is very large, more than twice that of the Pt sample in both the positive and negative directions, which is in agreement with the polarization current pattern recorded earlier.

Then, the test was performed with different pulse widths. The $V_{max}$ and pulse delay were set to 60 V and 1 ms, respectively, and the pulse width was taken as 0.5, 0.25, 0.125, 0.625 and 0.05 ms. It can be seen in Figure 9c,d that the remanent polarization increases with increasing pulse width for both samples. At small pulse widths, it is not enough to support a complete flip of polarization, resulting in a decrease in the remanent polarization value. Therefore, devices utilizing the ferroelectricity of AlScN thin films require a special design when setting the operating frequency. The remanent polarization, leakage currents and breakdown voltage, as well as the retention of the ferroelectricity, should be taken into account during the device and architecture design.

Finally, the $V_{max}$ and pulse width were kept constant and tested at pulse delays of 1, 10, 100, 1000 and 10,000 ms, respectively. It can be seen that the Pt sample results are smooth with no significant change, which is basically the same as the previous test. The remaining polarization value in the positive direction of the Mo sample also has no significant change, whereas the value in the negative direction gradually increases, as shown in Figure 9e,f. This means that the Mo sample has a larger polarization loss in the negative direction, which also corresponds to a larger leakage current in the negative direction. Therefore, AOGs on the film surface perpendicular to the c-axis can greatly compromise the ferroelectric properties. Moreover, multiple samples were tested to observe whether there is good consistency, as shown in Figure 9g,h. It can be seen that the negative polarization fluctuation of the Mo sample is slightly larger, and other points float in a small range.

Therefore, the abnormal grain orientation may seriously affect the ferroelectric properties of the films. It is possible that the abnormal grain orientation changes the original wurtzite structure near the interface between the metal and dielectric, and then affects the polarization properties of the film. From the test results, this effect is unidirectional and will greatly change the polarization characteristics in one direction. It can be reasonably speculated that, in addition to Mo, other metal materials may also bring different effects, which is worthy of further experimental verification.

## 4. Conclusions

In this paper, we analyzed the correlation between the ferroelectricity of aluminum scandium nitride and bottom metal electrodes. On one hand, the difference in crystal orientation of films grown on Pt or Mo metals will affect the ferroelectric properties. On the other hand, the inherent contact barrier between metal and dielectric materials will also affect ferroelectricity. The direction of driving voltage and frequency will also lead to different phenomena. After solving the problem of the leakage current, the high remnant polarization of more than 100 μC/cm^2^ and coercive field of 3 MV/cm exhibited by $Al_{0.7}Sc_{0.3}N$ films with good quality are suitable for ferroelectric memory devices. In FeRAM or FeFET, a high remnant polarization value can increase the storage density, while a suitable coercivity field can meet the storage window at a thin thickness. With further development, AlScN is expected to be widely used in commercial memory devices, as well as tunable RF applications.

## Data Availability

Data is available upon request.
